# Oncogenic potential of N-terminal deletion and S45Y mutant β-catenin in promoting hepatocellular carcinoma development in mice

**DOI:** 10.1186/s12885-018-4870-z

**Published:** 2018-11-12

**Authors:** Yu Qiao, Meng Xu, Junyan Tao, Li Che, Antonio Cigliano, Satdarshan P Monga, Diego F Calvisi, Xin Chen

**Affiliations:** 1 Department of Oncology, Beijing Hospital, National Center of Gerontology, Beijing, China; 2 Department of Bioengineering and Therapeutic Sciences and Liver Center, University of California, 513 Parnassus Avenue, San Francisco, CA 94143 USA; 3 Department of Hepatobiliary Surgery, The First Affiliated Hospital of Xi’an Jiaotong University, Xi’an, People’s Republic of China; 4 Department of Pathology, University of Pittsburgh School of Medicine, Pittsburgh, PA USA; 5 National Institute of Gastroenterology “S. de Bellis”, Research Hospital, Castellana Grotte, Italy; 6 Institute of Pathology, University Medicine Greifswald, Friedrich-Loeffler-Strasse 23e, 17489 Greifswald, Germany

**Keywords:** Hepatocellular carcinoma, β-Catenin, c-Met

## Abstract

**Background:**

Hepatocellular carcinoma (HCC) is one of the leading causes of cancer-related death worldwide with limited treatment options. Mutation of β-catenin is one of the most frequent genetic events along hepatocarcinogenesis. β-catenin mutations can be in the form of point mutation or large N-terminal deletion. Studies suggested that different β-catenin mutations might have distinct oncogenic potential.

**Methods:**

We tested the oncogenic activity of β-cateninS45Y, one of the most frequent point mutations of β-catenin, and ∆N90-β-catenin, a form of β-catenin with a large N-terminal deletion, in promoting HCC development in mice. Thus, we co-expressed β-cateninS45Y or ∆N90-β-catenin together with c-Met into the mouse liver using hydrodynamic injection.

**Results:**

We found that both β-catenin mutations were able to induce HCC formation in combination with c-Met at the same latency and efficiency. Tumors showed similar histological features and proliferation rates. However, immunohistochemistry showed predominantly nuclear staining of β-catenin in c-Met/∆N90-β-catenin HCC, but membrane immunoreactivity in c-Met/β-cateninS45Y HCC. qRT-PCR analysis demonstrated that both ∆N90-β-catenin and β-cateninS45Y induced the same effectors, although at somewhat different levels. In cultured cells, both ∆N90-β-catenin and β-cateninS45Y were capable of inducing TCF/LEF reporter expression, promoting proliferation, and inhibiting apoptosis.

**Conclusions:**

Our study suggests that β-cateninS45Y and ∆N90-β-catenin, in combination with the c-Met proto-oncogene, have similar oncogenic potential. Furthermore, nuclear staining of β-catenin does not always characterize β-catenin activity.

## Background

Hepatocellular carcinoma (HCC) is one of the most common cancers in the world, with up to 1 million cases diagnosed and almost 800,000 deaths globally each year [1]. Chronic infections by hepatitis B (HBV) and C (HCV) viruses, high alcohol consumption, obesity, type II diabetes, and exposure to aflatoxin B1 are the main risk factors for HCC. Treatment options for HCC are still very limited. For early stage HCC patients, surgical resection and liver transplantation are potentially curative. However, due to the lack of specific symptoms, most HCCs are diagnosed at late/unresectable stage. For these patients, the multi-kinase inhibitors Sorafenib and Regorafenib provide very limited survival benefits [2, 3]. Thus, there are major unmet medical needs for the treatment of HCC and elucidating the molecular mechanisms underlying HCC is of high importance to discover novel therapeutic approaches against this deadly disease.

Recent genomic studies provide a comprehensive genetic landscape of human HCCs. The most frequent genetic alterations found in this tumor type include mutations of telomerase reverse transcriptase (*TERT*) promoter region, *TP53*, *ARID1A*, *ARID2*, and *CTNNB1* genes. Among them, mutations of *CTNNB1*, which encodes β-catenin, were found in 13–43% of HCCs [4–6], suggesting that the Wnt/β-catenin cascade is a major driving oncogenic event along HCC development.

The Wnt/β-catenin signaling pathway regulates cell proliferation and differentiation, thus playing a critical role both in development and tumorigenesis [7]. In unstimulated cells, β-catenin predominantly locates at the plasma membrane as part of the adhesion junctions (AJs). The core of AJs consists of multiple cadherin family members, including E-Cadherin and α-catenin in addition to β-catenin [8]. The AJs have major roles in the stabilization of cell-cell adhesion and regulation of actin cytoskeleton. The cytoplasmic pool of β-catenin is maintained at low concentration via phosphorylation of its specific serine and threonine residues by a destruction complex. Activation of the Wnt pathway via its ligands results in the disruption of the complex, preventing β-catenin degradation. Subsequently, β-catenin translocates to the nucleus, where it interacts with T-cell factor (TCF)/lymphoid enhancer factor (LEF) transcription factors, leading to the upregulation of various target genes, such as glutamine synthetase (GS), c-Myc, cyclin D1, G-protein coupled receptor 5 (LGR5), and Axin2 [9–11]. Therefore, β-catenin may have distinct roles depending on its localization, namely at the cell membrane as part of AJs, and in the nucleus to induce downstream gene expression. β-catenin nucleus translocation is considered a major mechanism of activating the Wnt/β-catenin cascade in cells.

As mutations of β-catenin are one of the most frequent genetic events during HCC development and progression, they have been extensively studied. In particular, it has been found that HCCs harboring β-catenin mutations tend to occur more frequently in older patients, especially those who are chronically infected by HBV. In addition, β-catenin mutant HCCs mainly develop in the noncirrhotic liver, often are well differentiated, and are associated with better prognosis [12, 13]. Most β-catenin mutations in human HCC occur in exon 3 at serine/threonine sites and their neighboring amino acids. In addition, large exon 3 deletions could also be found. All these mutations prevent the phosphorylation and subsequent degradation of β-catenin protein, leading to the ligand-independent activation of the Wnt/β-catenin cascade. In a recent study, it was found that β-catenin mutations may have different activities: large in-frame exon 3 deletions and point mutations located at the D32-S37 residues can induce strong β-catenin activity, point mutations located at T41 can result in a moderate activity, and point mutations located at S45 may be responsible for a relative weak activity [11].

The c-Met protooncogene encodes a tyrosine kinase-type growth factor receptor with an affinity for hepatocyte growth factor (HGF). Activation of c-Met is frequently found in human HCCs [14]. Recently, we demonstrated that concomitant activation of c-Met and β-catenin mutations could be identified in a subset of human HCCs [15]. Also, we showed that co-expression of human c-Met and a point-mutant-β-catenin (β-cateninS45Y) via hydrodynamic transfection leads to HCC formation in mice. Gene expression analysis confirmed that these mouse tumors mimic the subset of human HCCs with c-Met activation and β-catenin mutations [15].

In the present study, we investigated the oncogenic potential of the weak β-catenin mutation (β-cateninS45Y) and the strong β-catenin mutation (∆N90-β-catenin) in inducing HCC formation in mice. Our investigation demonstrates that both alterations can equally promote HCC development in combination with the c-Met protooncogene.

## Methods

### Cell lines and culture

Two mouse HCC cell lines, HCC3–4 and HCC4–4, which were derived from c-Myc transgenic mice [16], were cultured in Dulbecco’s modified Eagle medium (DMEM) supplemented with 10% FBS, penicillin (100 U/mL) and streptomycin (100 μg/mL) in a humidified 5% CO2 incubator at 37 °C.

### Constructs and reagents

The constructs used for mouse injection including pT3-EF1α, pT3-EF1α-c-Met, pT3-EF1α-β-cateninS45Y (with N-terminal Myc tag), pT3-EF1α-∆N90-β-catenin (with N-terminal Myc tag), and pCMV/sleeping beauty transposase (pCMV/SB) have been described previously [15, 17]. All the plasmids used for in vivo experiments were purified using the Endotoxin free Maxi prep kit (Sigma-Aldrich, St.Louis, MO) before being injected into the mice. Super 8× TopFlash and Super 8× FopFlash plasmids were obtained from Addgene (plasmid 12,456, plasmid 12,457); and pRL-CMV Renilla luciferase plasmid was purchased from Promega (Madison, WI).

### Mice and hydrodynamic tail injection

Wild-type (WT) FVB/N mice were obtained from The Jackson Laboratory (Bar Harbor, Maine, USA). Hydrodynamic injection was performed as described previously [18]. In brief, 20 μg pT3-EF1α-β-cateninS45Y or pT3-EF1α-∆N90-β-catenin was mixed with 20 μg pT3-EF1α-c-Met along with 1.6 μg pCMV/SB in 2 ml of normal saline (0.9%NaCl). The solution was filtered through 0.22 μm filter (EMD Millipore, Burlington, MA), and injected into the lateral tail vein of 6 to 8-week-old FVB/N mice in 5 to 7 s. Mice were housed, fed, and monitored in accord with protocols approved by the Committee for Animal Research at the University of California San Francisco (San Francisco, CA). All animals were carefully monitored for signs of morbidity or discomfort. Close attention was paid to the abdominal girth. Animals were sacrificed 7 weeks after injection. Body and liver weights were recorded.

### Immunohistochemical staining

Mouse liver lesions were fixed in 4% paraformaldehyde overnight at 4 °C and embedded in paraffin. Hematoxylin & Eosin (H&E) staining on 4 μm liver sections was performed to determine the time of appearance and characteristics of neoplastic foci. For immunohistochemistry, antigen retrieval was performed in 10 mM sodium citrate buffer (pH 6.0) by placement in a microwave oven on high for 10 min, followed by a 20-min cool down at room temperature. After a blocking step with the 5% goat serum and Avidin-Biotin blocking kit (Vector Laboratories Inc., Burlingame, CA), the slides were incubated with the following primary antibodies: mouse anti-β-catenin (1:200; BD Transduction Laboratories™, San Jose, CA), mouse anti-Glutamine Synthetase (1:500; BD Transduction Laboratories™), β-catenin (D10A8) XP® Rabbit mAb (1:100; Cell Signaling Technology, Danvers, MA), anti-Myc tag (1:1000; Maine Medical Center Research Institute, Scarborough, ME), Ki-67 (1:150; Santa Cruz Biotechnology, Santa Cruz, CA) overnight at 4 °C. Slides were then subjected to 3% hydrogen peroxide for 10 min to quench endogenous peroxidase activity and subsequently the biotin conjugated secondary antibody was applied at a 1:500 dilution for 30 min at room temperature. Signal was detected using the Vectastain ABC Elite kit (Vector Laboratories Inc.) and developed using DAB (Vector Laboratories, Inc.). Sections were counterstained with hematoxylin solution (ThermoFisher Scientific, Pittsburgh, PA) and passed through the dehydration process and covered-slipped.

### Western blot analysis

Tissue were homogenized with a Polytron and cells were washed in PBS and lysed in M-PER™ Mammalian Protein Extraction Reagent (ThermoFisher Scientific) containing the Halt™ Protease Inhibitor Cocktail (ThermoFisher Scientific). Protein concentration was quantified using the Pierce™ Microplate BCA Protein Assay Kit (ThermoFisher Scientific). Membranes were blocked in in 5% non-fat milk for 1 h and incubated with the following primary antibodies: β-catenin (1:2000; BD Transduction Laboratories™), p-β-catenin (Ser45; 1:1000; Cell Signaling Technology), Myc-tag (1:2000; MMCRI), Glutamine Synthetase (1:5000; BD Transduction Laboratories), c-Met (1:400, Invitrogen), p-Met (Tyr1234/1235; 1:1000; Cell Signaling Technology), cyclin D1 (1:10000; Abcam, Cambridge, UK), β-actin (1:5000; Sigma-Aldrich) and GAPDH (1:10000; EMD Millipore). Each primary antibody was followed by incubation with secondary antibody (1:5000; Jackson ImmunoResearch Laboratories Inc., West Grove, PA) for 1 h. After appropriate washing, bands were revealed with the Super Signal West Dura Kit (ThermoFisher Scientific).

### Quantitative real-time reverse-transcription polymerase chain reaction

Total RNAs were isolated from frozen mouse tissue samples using the Quick-RNA™ MiniPrep (Zymo Research, Irvine, CA). Reverse transcription was conducted according to the manufacturer’s instructions (Invitrogen, Carlsbad, CA). The mouse primers used for PCR analysis were synthesized by Integrated DNA Technologies (Coralville, IA). The sequences of the primers were as follows: ribosomal RNA (rRNA):5’-CGGCTACCACATCCAAGGAA-3′ (Forward) and 5’-GCTGGAATTACCGCGGCT-3′ (Reverse); Alpha Fetoprotein (AFP): 5’-TCTGCTGGCACGCAAGAAG-3′ (Forward) and 5’-TCGGCAGGTTCTGGAAACTG-3′ (Reverse); Glypican 3 (GPC3): 5’-CAGCCCGGACTCAAATGGG-3′ (Forward) and 5’-CAGCCGTGCTGTTAGTTGGTA-3′ (Reverse); axis inhibition protein 2 (AXIN2): 5’-GCTCCAGAAGATCACAAAGAGC-3′ (Forward) and 5’-AGCTTTGAGCCTTCAGCATC-3′ (Reverse); G-protein coupled receptor 5 (LGR5): 5’-ACCGAGCCTTACAGAGCCT-3′ (Forward) and 5’-GCCGTCGTCTTTATTCCATTGG-3′ (Reverse); MYC proto-oncogene (c-Myc): 5’-TGTACCTCGTCCGATTCC-3′ (Forward) and 5’-CATCTTCTTGCTCTTCTTCAG-3′ (Reverse); Cyclin D1 (CCND1): 5’-CGTGGCCTCTAAGATGAAGGA-3′ (Forward) and 5’-CCTCGGGCCGGATAGAGTAG-3′ (Reverse); Glutamine Synthetase (GS): 5’-CAGGCTGCCATACCAACTTCA-3′ (Forward) and 5’-TCCTCAATGCACTTCAGACCAT-3′ (Reverse); T-Box3 (TBX3): 5’-CAGGCAGCCTTCAACTGCTT-3′ (Forward) and 5’-GGACACAGATCTTTGAGGTTGGA-3′ (Reverse); Ornithine Aminotransferase (OAT): 5’-GGAGTCCACACCTCAGTCG-3′ (Forward) and 5’-CCACATCCCACATATAAATGCCT-3′ (Reverse); Leukocyte cell-derived chemotaxin-2 (LECT2): 5’-CCCACAACAATCCTCATTTCAGC-3′ (Forward) and 5’-ACACCTGGGTGATGCCTTTG-3′ (Reverse). Quantitative real-time polymerase chain reaction was performed with 100 ng of cDNA, using an ABI Prism 7000 Sequence Detection System and TaqMan Universal PCR Master Mix (ThermoFisher Scientific). Cycling conditions were: 10 min of denaturation at 95 °C and 40 cycles at 95 °C for 15 s and at 52 °C for 1 min. Quantitative values were calculated by using the PE Biosystems Analysis software and expressed as N target (NT). NT = 2^-ΔCt^, wherein the ΔCt value of each sample was calculated by subtracting the maximum Ct value of the target gene from the average Ct value of the rRNA gene.

### Dual-luciferase reporter assay

HCC3–4 and HCC4–4 cells were plated in triplicate in 24-well plate at 80–90% confluency. Cells were transfected using the Lipofectamine 2000 reagents (Invitrogen). In brief, each well was transfected with 600 ng of pT3-EF1α (empty vector control) or pT3-EF1α-β-cateninS45Y or pT3-EF1α-∆N90-β-catenin, together with 200 ng of TOPFlash plasmid DNA or the negative control FOPFlash, as well as 8 ng of pRL-CMV. Cells were harvested 48 h post transfection. Luciferase activity was measured using the Dual-Luciferase® Reporter Assay System (Promega), according to the manufacturer’s protocol. Experiments were repeated at least three times in triplicate.

### Assessment of proliferation and apoptosis

HCC3–4 and HCC4–4 cells were plated in triplicate in 24-well plate at 80–90% confluency. Cells were transfected using the Lipofectamine 2000 reagents (Invitrogen). Proliferation and apoptosis were assessed using the BrdU Cell Proliferation Reagent (Cell Signaling Technology) and the Cell Death Detection Elisa Plus Kit (Roche Molecular Biochemicals, Indianapolis, IN), respectively, following the manufacturers’ protocol. Experiments were repeated at least three times in triplicate.

### Statistical analysis

Statistical analysis was performed using Student’s t-test and Tukey-Kramer test. The data were expressed as the mean ± SD of at least three independent experiments. *P* < 0.05 was considered significant.

## Results

### β-cateninS45Y and ∆N90-β-catenin cooperate with c-Met to induce HCC formation in mice

**Fig. 1:**
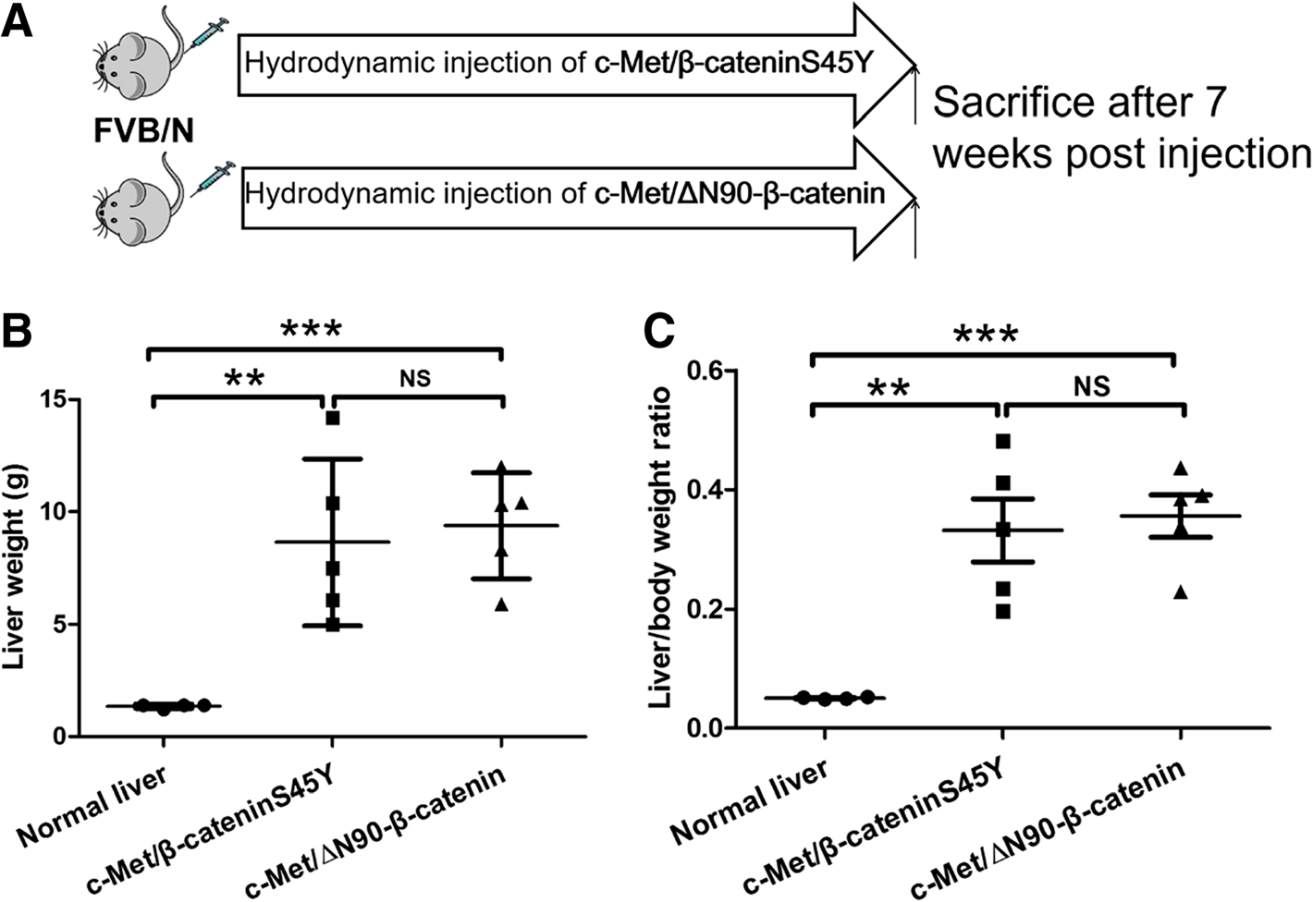
β-catenin mutants cooperate with c-Met to promoter HCC development in mice. **a** Study design. **b** and **c** Liver weight and liver/body weight ratio among wild-type mice, c-Met/β-cateninS45Y mice, and c-Met/∆N90-β-catenin mice. Data are presented as mean ± standard deviation. Student’s t-test: **: *P* < 0.01, ***: *P* < 0.001; NS: Not significant

First, we investigated the oncogenic potential of “weak” β-catenin mutant (S45Y) and “strong” β-catenin mutant (∆N90-β-catenin) proto-oncogenes. However, since overexpression of β-cateninS45Y or ∆N90-β-catenin alone does not lead to tumor formation [15, 17, 19], we hydrodynamically injected β-cateninS45Y or ∆N90-β-catenin (both with a Myc tag) together with c-Met, a proto-oncogene frequently overexpressed in human HCC, whose single overexpression does not suffice for HCC development in mice [14], in the mouse liver. The mice receiving the combination of plasmids were referred to as c-Met/β-cateninS45Y and c-Met/∆N90-β-catenin, respectively. All c-Met/β-cateninS45Y or c-Met/∆N90-β-catenin mice were harvested after 7 weeks post injection (Fig. 1a). Upon dissection, all c-Met/β-cateninS45 and c-Met/∆N90-β-catenin injected mice developed liver tumors (Table 1). Using total liver weight as well as liver/body weight ratio as the measurement of tumor burden, all c-Met/β-cateninS45Y or c-Met/∆N90-β-catenin injected mice had significantly increased tumor burden when compared to wild-type mice (Fig. 1b and c), whereas the overall tumor burden was similar between c-Met/β-cateninS45Y and c-Met/∆N90-β-catenin mouse cohorts (Fig. 1b and c).

**Table 1:** Detailed mouse data from c-Met/β-cateninS45Y and c-Met/ΔN90-β-catenin injected mice

Mice	Harvest time point (weeks post injection)	Liver phenotype	Body weight (g)	Liver weight (g)	Liver/body ratio (%)
c-Met/β-cateninS45Y	7	Big tumor	25.5	5.0	19.6078
c-Met/β-cateninS45Y	7	Big tumor	29.5	14.2	48.1356
c-Met/β-cateninS45Y	7	Big tumor	25.3	10.4	41.1067
c-Met/β-cateninS45Y	7	Big tumor	22.5	7.5	33.3333
c-Met/β-cateninS45Y	7	Big tumor	26.0	6.1	23.4615
c-Met/ΔN90-β-catenin	7	Big tumor	26.4	10.3	39.0152
c-Met/ΔN90-β-catenin	7	Big tumor	27.1	10.4	38.3764
c-Met/ΔN90-β-catenin	7	Big tumor	24.6	8.3	33.7398
c-Met/ΔN90-β-catenin	7	Big tumor	27.5	12.0	43.6364
c-Met/ΔN90-β-catenin	7	Big tumor	25.8	5.9	22.8682

**Fig. 2:**
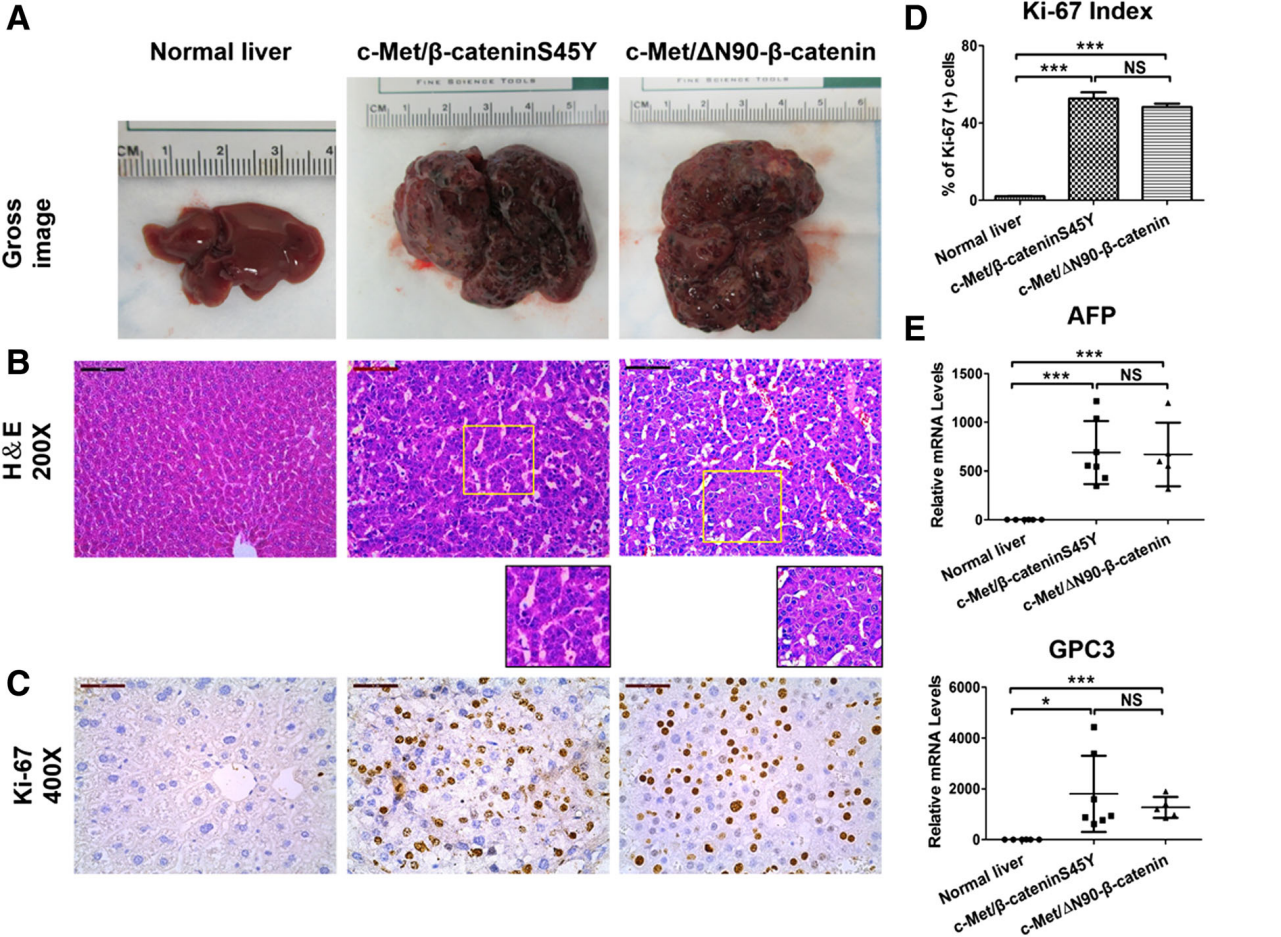
Histological features of normal liver as well as HCCs from c-Met/β-cateninS45Y and c-Met/∆N90-β-catenin injected mice. qRT-PCR analysis of HCC tumor markers (**a**) Gross images. **b** H&E staining; scale bar = 200 μm. **c** Ki-67 IHC images; scale bar = 100 μm. **d** Quantification of percentage of Ki-67 (+) cells in normal liver and mouse HCC lesions. **e** qRT-PCR analysis of HCC tumor markers AFP and GPC3 in normal liver and mouse HCC lesions. Student’s t-test: *: *P* < 0.05; ***: *P* < 0.001; NS: Not significant

Grossly, numerous tumor nodules could be found throughout the liver parenchyma of c-Met/β-cateninS45Y and c-Met/∆N90-β-catenin mice (Fig. 2a). Histologically, H&E staining of representative liver sections from c-Met/β-cateninS45Y and c-Met/∆N90-β-catenin mice showed the presence of well-differentiated HCC with solid or macro-trabecular growth pattern (Fig. 2b). Tumor cells were highly proliferative as revealed via immunohistochemical (IHC) staining of Ki-67, whereas Ki-67 positive cells were rarely found in normal liver (Fig. 2c). Quantification of Ki-67 IHC demonstrated that c-Met/β-cateninS45Y and c-Met/∆N90-β-catenin HCC had similar cell proliferation rates (Fig. 2d). Finally, qRT-PCR analysis of HCC tumor markers AFP and GPC3 showed that both HCC samples had higher expression of these genes than normal liver, and there was no difference between the two mouse HCC cohorts (Fig. 2e).

Collectively, our results suggest that β-cateninS45Y and ∆N90-β-catenin cooperate with c-Met to promote HCC formation in vivo at similar latency and efficiency.

### Different β-catenin staining patterns between c-Met/∆N90-β-catenin and c-Met/β-cateninS45Y mouse HCC

**Fig. 3:**
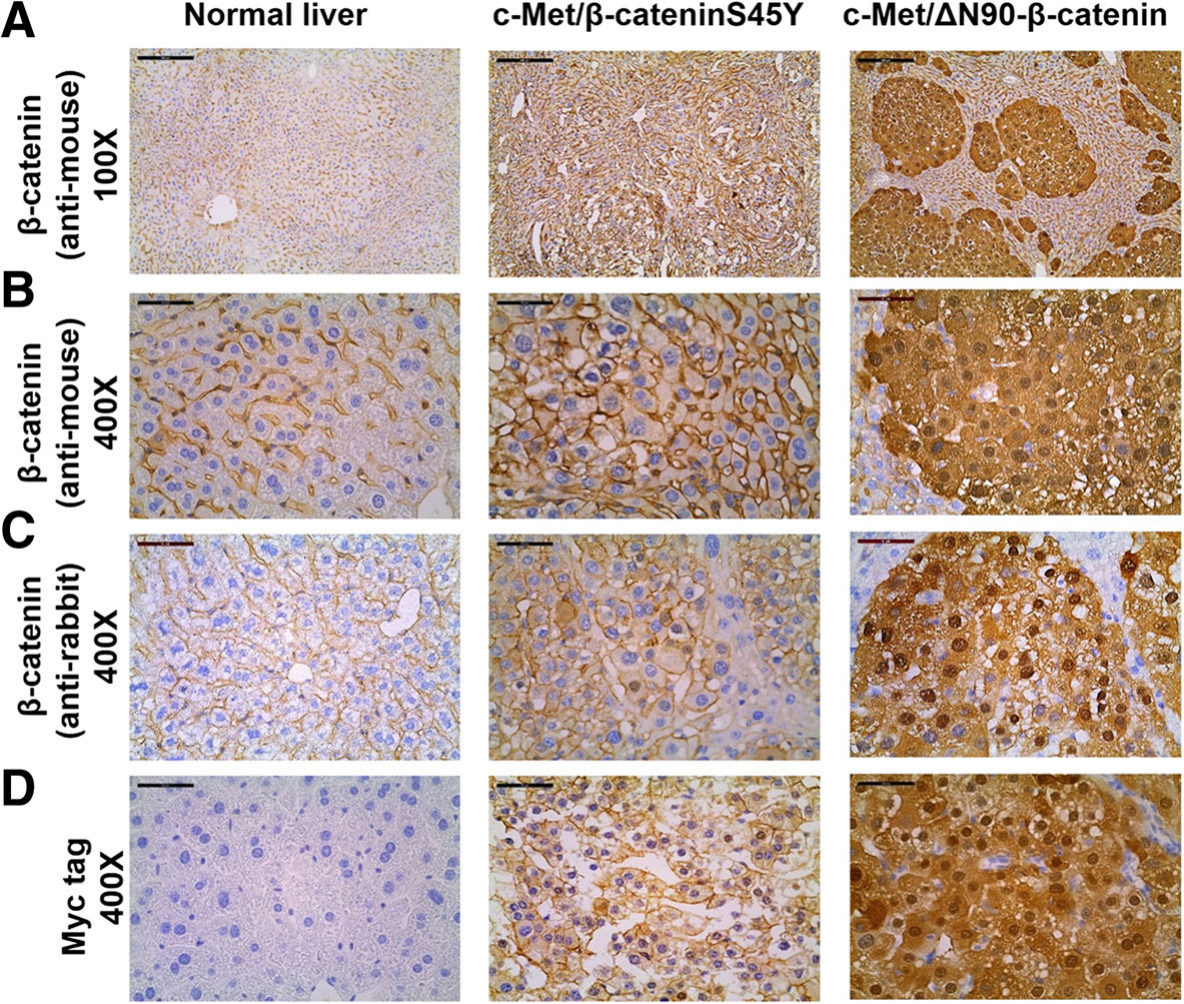
Immunohistochemical patterns of β-catenin in normal liver and in HCC lesions of c-Met/β-cateninS45Y c-Met/∆N90-β-catenin mice. **a** and **b** Immunohistochemistry (IHC) of β-catenin using a mouse monoclonal antibody; scale bar = 400 μm in A; and 100 μm in B; (**c**) IHC of β-catenin using a rabbit monoclonal antibody; scale bar = 100 μm; (**d**) IHC of ectopically injected β-catenin using an anti-Myc tag antibody; scale bar = 100 μm

Next, we performed IHC of β-catenin in normal liver as well as HCCs from c-Met/β-cateninS45Y and c-Met/∆N90-β-catenin mice. Using a widely used mouse monoclonal antibody against β-catenin, we found that, in normal liver, β-catenin staining could only be detected at the hepatocyte membrane (Fig. 3a and b). In c-Met/∆N90-β-catenin HCC lesions, instead, strong nuclear and cytoplasm staining of β-catenin could be readily appreciated. Surprisingly, c-Met/β-cateninS45Y HCCs showed predominantly intense membranous and very weak cytoplasmic β-catenin staining (Fig. 3a and b). To rule out that this event may be due to the antibody applied, we stained another set of slides using a rabbit monoclonal antibody against β-catenin. Of note, the same results were obtained using the second anti-β-catenin antibody (Fig. 3c). Furthermore, as the ectopically injected β-catenin construct has a N-terminal Myc tag, we stained tumor tissue specimens with an antibody against Myc tag. Again, we found strong nuclear/cytoplasmic staining pattern of β-catenin in c-Met/∆N90-β-catenin HCC lesions, while intense membranous immunoreactivity of β-catenin characterized c-Met/β-cateninS45Y HCC lesions (Fig. 3d). As expected, no staining for Myc tag was detected in the un-injected normal mouse liver tissue, supporting the specificity of the anti-tag antibody (Fig. 3d).

**Fig. 4:**
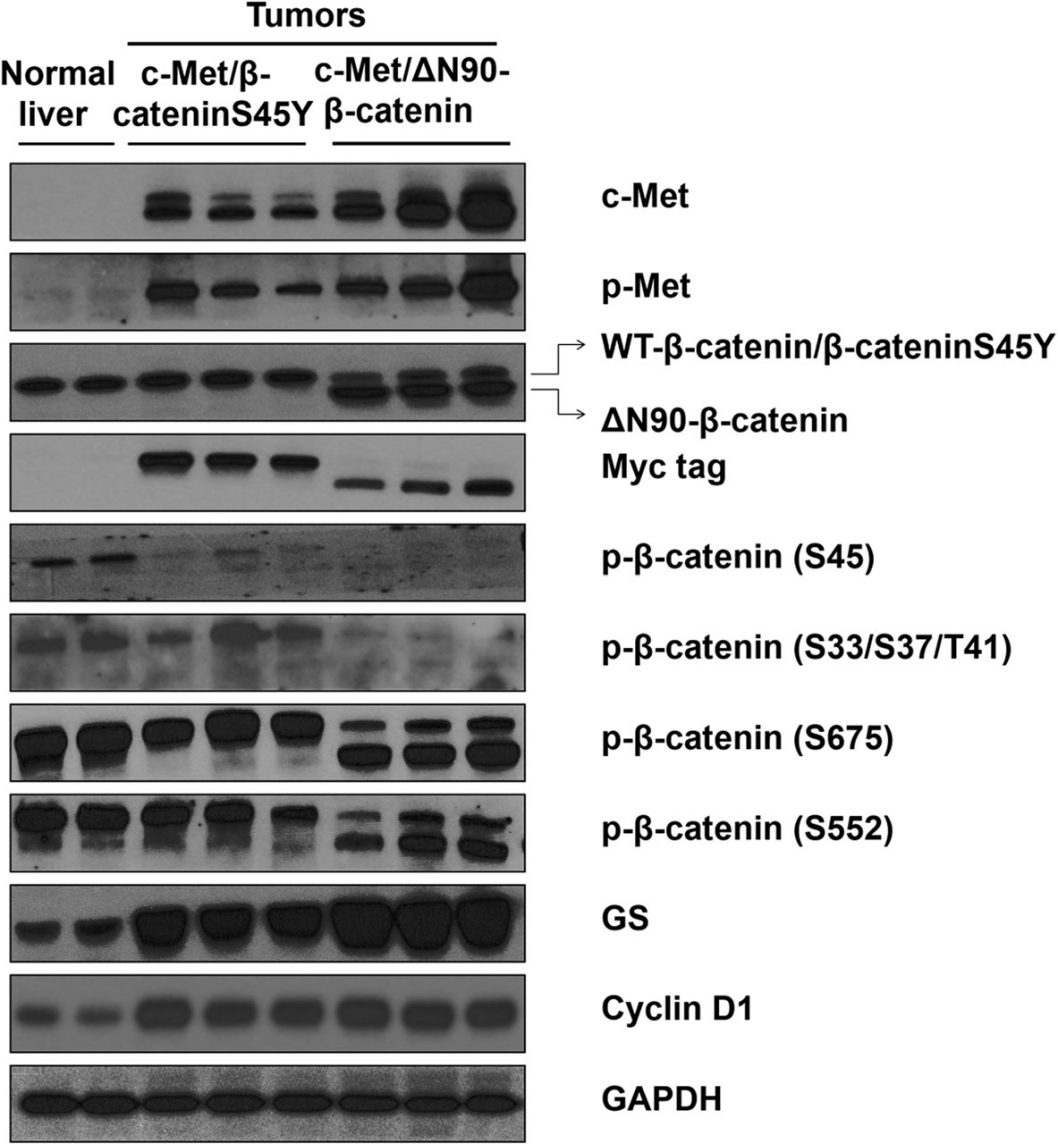
Biochemical analysis of HCC lesions of c-Met/β-cateninS45Y c-Met/∆N90-β-catenin mice. Western blot analysis of c-Met, p-Met, β-catenin, Myc tag, p-β-catenin (S45), p-β-catenin (S33/S37/T41), p-β-catenin (S675), p-β-catenin (S552), GS, and cyclin D1 in normal liver as well as HCC lesions from c-Met/β-cateninS45Y c-Met/∆N90-β-catenin mice. GAPDH were used as a loading control. Please note the presence of two bands for the β-catenin protein. The upper band represents endogenous β-catenin protein or β-cateninS45Y protein. The lower band represents ectopically injected ∆N90-β-catenin protein

At the molecular level, using Western blotting, we found that both c-Met/β-cateninS45Y and c-Met/∆N90-β-catenin HCC lesions showed expression of ectopically injected human c-Met (Fig. 4), and c-Met was activated as demonstrated by the high phosphorylated(p)-Met levels. Ectopically expressed β-catenin could be detected via Western blotting using an anti-β-catenin antibody as well as a Myc tag antibody (Fig. 4). Phosphorylated β-catenin at Ser45 could only be found in normal liver tissues; and p-β-catenin at S33/S37/T41 was lost in c-Met/∆N90-β-catenin HCCs (Fig. 4). Phosphorylation of β-catenin at its C-terminal Ser552 and Ser675 residues was detected in the endogenous and exogenous β-catenin proteins (Fig. 4).

**Fig. 5:**
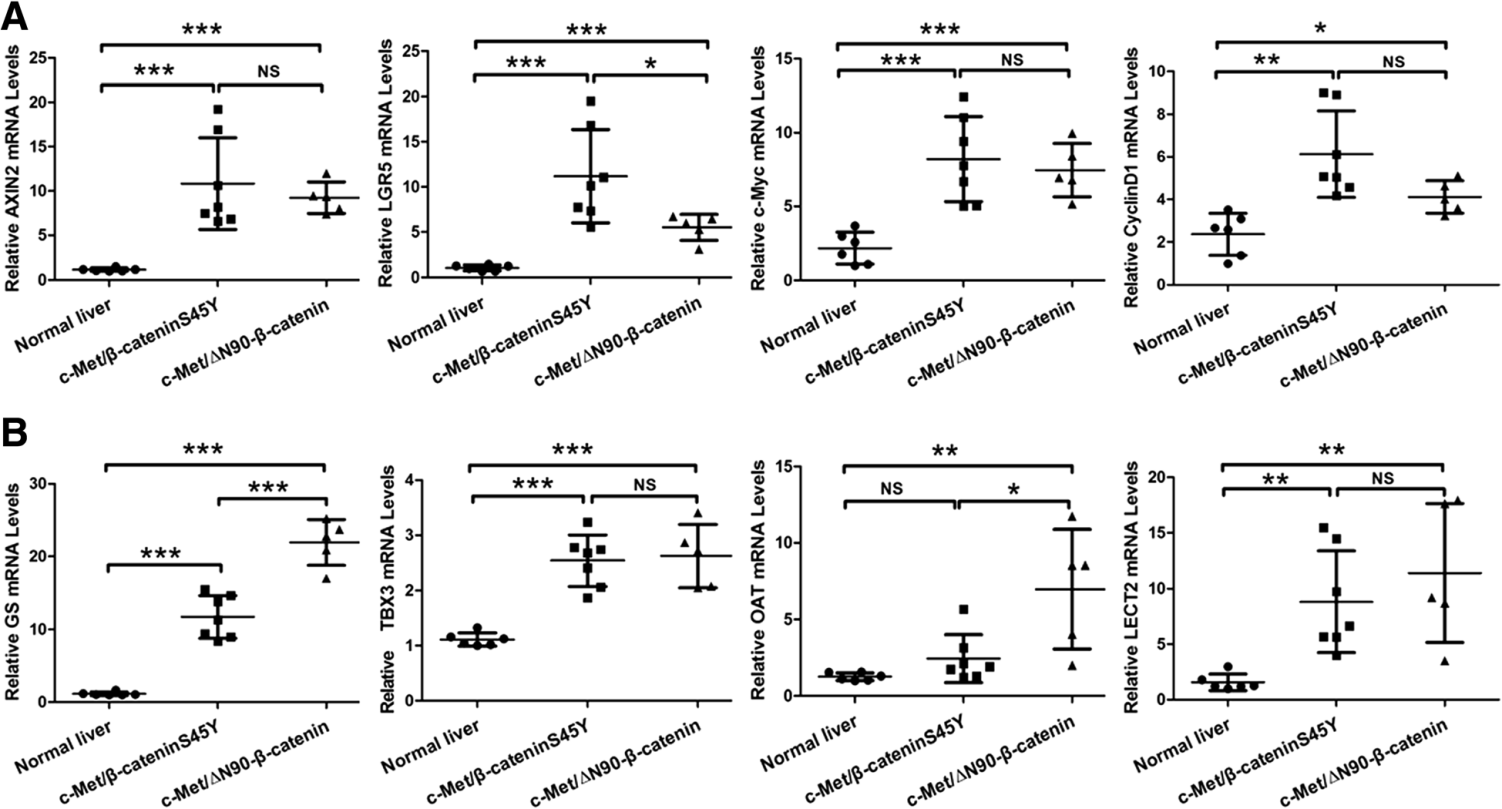
qRT-PCR analysis of β-catenin target gene expression in c-Met/∆N90-β-catenin and c-Met/β-cateninS45Y mouse HCC samples. **a** qRT-PCR analysis of mRNA levels of pan-β-catenin target genes: AXIN2, LGR5, c-Myc and cyclin D1. **b** qRT-PCR analysis of mRNA levels of liver specific β-catenin target genes: GS, TBX3, OAT, and LECT2. Student’s t-test: *P < 0.05; **P < 0.01; ***P < 0.001

Next, we investigated whether c-Met/β-cateninS45Y and c-Met/∆N90-β-catenin mouse HCCs showed similar expression patterns of Wnt/β-catenin canonical target genes. A total of 8 genes were analyzed via qRT-PCR, including the pan-β-catenin targets Axin2, LGR5, c-Myc, and Cyclin D1 (Fig. 5a), as well as liver specific β-catenin targets GS, TBX3, OAT and LECT2 (Fig. 5b). We found that overall HCC lesions from c-Met/β-cateninS45Y and c-Met/∆N90-β-catenin mice exhibited higher expression of β-catenin target genes than normal liver, and the two HCC sample cohorts had similar expression patterns (Fig. 5a and b). However, some subtle differences were noted. Specifically, by qRT-PCR, we found that the pan-β-catenin target LGR5 expression was higher in HCCs from c-Met/β-cateninS45Y mice than that in c-Met/∆N90-β-catenin mice. On the other hand, liver specific β-catenin targets GS and OAT were expressed at highest levels in c-Met/∆N90-β-catenin HCC mouse lesions. Western blotting confirmed the higher expression of GS in c-Met/∆N90-β-catenin tumors and similar expression of Cyclin D1 in c-Met/β-cateninS45Y and c-Met/∆N90-β-catenin mouse HCCs (Fig. 4).

In summary, our study shows that β-catenin displays distinct staining patterns in c-Met/β-cateninS45Y and c-Met/∆N90-β-catenin mouse HCCs. However, the staining patterns were not associated with β-catenin activity, as both c-Met/β-cateninS45Y and c-Met/∆N90-β-catenin mouse HCCs demonstrated high and similar expression of β-catenin target genes.

### ∆N90-β-catenin and β-cateninS45Y induce TCF/LEF reporter expression in mouse HCC cell lines

**Fig. 6:**
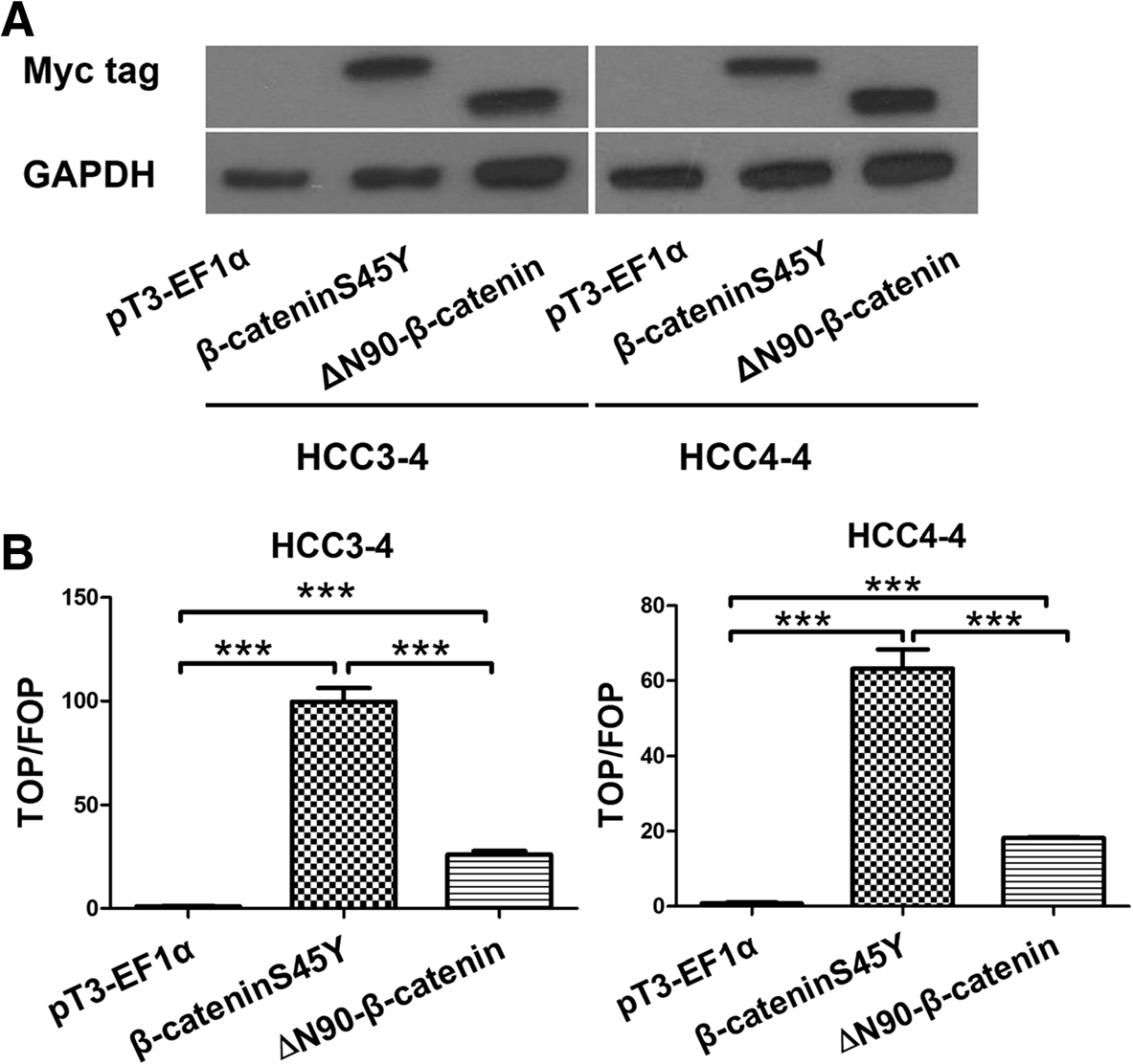
β-cateninS45Y and ∆N90-β-catenin mutants activate TCF/LEF reporter in mouse HCC cell lines. **a** Western blot analysis confirmed the expression of transfected β-cateninS45Y or ∆N90-β-catenin plasmids in HCC3–4 and HCC4–4 mouse HCC cell lines. **b** TCF/LEF reporter activity in HCC3–4 and HCC4–4 mouse HCC cell lines following the transfection of pT3-EF1α-β-cateninS45Y and pT3-EF1α-∆N90-β-catenin, as analyzed using TopFlash/FopFlash reporter assays. Student’s t-test: ***P < 0.001

Subsequently, we evaluated the transcriptional activity of β-cateninS45Y versus ∆N90-β-catenin with the widely used TCF/LEF reporter constructs. Specifically, two mouse HCC cell lines isolated from c-Myc transgenic mice, HCC3–4 and HCC4–4 cells [16], were used. These cells have high expression of human c-Myc and no basal activation of the Wnt/β-catenin signaling. TopFlash and FopFlash reporter assays were carried out in these two cell lines. Western blotting proved the successful expression of transfected β-cateninS45Y and ∆N90-β-catenin plasmids (Fig. 6a). The ratio of TOP/FOP was significantly increased following the transfection of pT3-EF1α-β-cateninS45Y and pT3-EF1α-∆N90-β-catenin compared with control pT3-EF1α empty vector (Fig. 6b). In addition, β-cateninS45Y mutant showed significantly higher TCF/LEF reporter activity than ∆N90-β-catenin mutant.

**Fig. 7:**
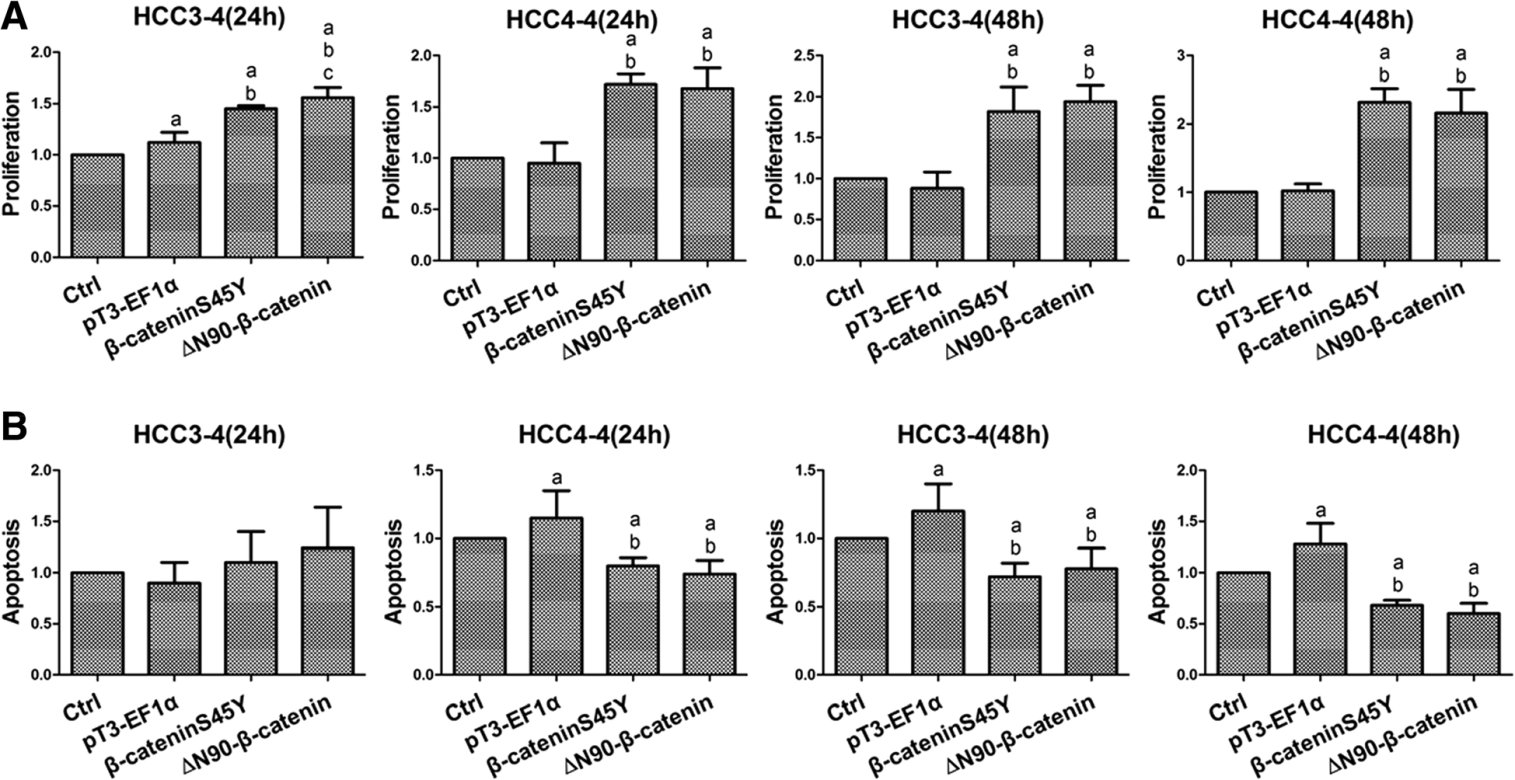
β-cateninS45Y and ∆N90-β-catenin mutants similarly affect proliferation and apoptosis in mouse HCC cell lines. Relative proliferation and apoptosis rates in HCC3–4 and HCC4–4 cell lines after transfection of the two β-catenin mutant forms at different time points (24 and 48 h). **a** proliferation; (**b**) apoptosis. Data are presented as mean ± SD. a. vs control (untransfected cells); b. vs pT3-EF1α; c. vs pT3-EF1α-β-cateninS45Y

Finally, we assessed the consequence of the transfection of β-cateninS45Y and ∆N90-β-catenin constructs in terms of proliferation and apoptosis in HCC3–4 and HCC4–4 cells. Noticeably, both mutant forms of β-catenin were able to induce higher proliferation and lower apoptosis than untreated and empty vector transfected cells (Fig. 7). Once again, no significant differences were detected for proliferation and apoptosis between β-cateninS45Y- and ∆N90-β-catenin-transfected cells (Fig. 7).

## Discussion

Somatic mutations of β-catenin are one of the most frequent genetic alterations along hepatocarcinogenesis. Specifically, in the most recently released TCGA dataset based on whole-exome sequencing of 363 HCC cases, β-catenin mutation was identified in 27% cases [4]. Survey of COSMIC database reveals that in 5306 published HCC cases, β-catenin mutations were detected in 18.22% or 967 cases [20]. Overall, these results demonstrate the β-catenin mutation is the third most frequent somatic alteration in HCC. Importantly, in a recent study, Torrecilla et al. analyzed the clonal evolution of human HCCs by characterizing the mutation spectrum of early stage and late stage HCC lesions [21]. β-catenin mutation was identified as one of the three trunk driver genetic mutations, together with *TERT* promoter mutation and *TP53* mutation, in HCCs [21]. Trunk mutations refer to those genetic alterations that occur early during tumor evolution, function as early drivers for tumorigenesis [22] and are present in every tumor subclone. These trunk mutations may therefore constitute the most robust and reliable therapeutic targets for cancer treatment [22]. Thus, it is critical to understand the oncogenic potential of trunk mutations, in this case β-catenin mutations, in order to search for therapies that may lead to synthetic lethality in combination with β-catenin mutations for HCC treatment.

In this study, we compared the oncogenic potential of β-cateninS45Y and ∆N90-β-catenin mutations in combination with c-Met proto-oncogene in promoting HCC development using mouse modeling. In a previous study, β-cateninS45Y was characterized as a “weak” mutant, whereas ∆N90-β-catenin was characterized as a “strong” mutant [11]. We found that at least when overexpressed in the mouse liver, β-cateninS45Y and ∆N90-β-catenin, together with c-Met, are capable of inducing HCC development at the same rate and latency. Histological and molecular analysis revealed that HCCs induced by two mutant forms of β-catenin are highly similar. Some subtle difference in terms of gene expression patterns was noted. GS expression has been used clinically as the measurement of activated β-catenin in human HCCs. In the present investigation, expression of GS was induced at higher levels in ∆N90-β-catenin/c-Met HCC than β-cateninS45Y/c-Met mouse HCC. Of note, in human hepatic adenoma and HCC samples, GS staining was found to be ubiquitously strong in tumor samples with large deletion of β-catenin, whereas human hepatic adenoma and HCC samples harboring β-cateninS45Y mutation showed a diffusely heterogeneous GS staining pattern [11]. At mRNA level, GS expression tends to be higher in human HCCs with “strong” β-catenin mutations than β-cateninS45Y mutant [11]. Taken together, the results support the hypothesis that different β-catenin mutations may induce different levels of GS expression in mouse and human HCCs.

Perhaps the most intriguing results from our current study is that ∆N90-β-catenin/c-Met mouse HCC demonstrated strong nuclear β-catenin staining while β-cateninS45Y/c-Met showed predominantly membranous β-catenin immunolabeling (Fig. 3). It is worth to note that β-catenin may only require transient nuclear translocation to induce downstream gene expression. One such example is that in the normal liver, a layer of pericentral hepatocytes exhibits activated β-catenin dependent expression of GS, but only membrane localization of β-catenin is observed [23]. In human HCCs, tumors with β-catenin mutations show either nuclear or membranous staining patterns. Therefore, nuclear β-catenin is not a reliable indicator of β-catenin mutation status in both mouse and human HCC samples. Other, markers, such as IHC of GS or qRT-PCR of Wnt/β-catenin target genes should be used instead to measure Wnt/β-catenin activity in HCC.

Previous studies showed that approximately 10% of human HCCs have concomitant β-catenin mutations along with c-Met overexpression [15]. β-cateninS45Y/c-Met mouse HCC model has been shown to share similar gene expression patterns with this subset of human HCCs [15]. Thus, the murine models with β-catenin mutations and c-Met overexpression are excellent preclinical systems to characterize novel targeted therapy against HCC. Our investigation suggests that β-cateninS45Y/c-Met and ∆N90-β-catenin/c-Met are equivalent in inducing HCC formation in mice. Therefore, both models could be effectively used as a preclinical tool to study the efficacy of new therapeutic approaches against this deadly disease.

## Conclusions

Our study suggests that β-cateninS45Y and ∆N90-β-catenin have similar oncogenic potential. Furthermore, nuclear staining of β-catenin does not always characterize β-catenin activity.

## Abbreviations

AFP: Alpha fetoprotein
AXIN2: Axis inhibition protein 2
CCND1: Cyclin D1
c-Myc: Myc proto-oncogene
DMEM: Dulbecco’s modified Eagle medium
GPC3: Glypican 3
GS: Glutamine synthetase
H&E: Hematoxylin & eosin
HBV: Hepatitis B viruses
HCC: Hepatocellular carcinoma
HCV: Hepatitis C viruses
HGF: Hepatocyte growth factor
IHC: Immunohistochemistry
LECT2: Leukocyte cell-derived chemotaxin-2
LEF: Lymphoid enhancer factor
LGR5: G-protein coupled receptor
OAT: Ornithine Aminotransferase
qRT-PCR: Quantitative real-time reverse-transcription polymerase chain reaction
SB: Sleeping beauty transposase
TBX3: T-Box3
TCF: T-cell factor
*TERT*: Telomerase reverse transcriptase
